# Report of the Trial of the People vs. Dr. Horatio N. Loomis, for Libel

**Published:** 1850-09

**Authors:** 


*Report of the Trial of the People* vs. *Dr. Horatio N. Loomis, for Libel.* Tried at the Erie County Oyer and Terminer, June 24th, 1850. Justice Mullet, presiding; John Treanor and Leander J. Roberts, Associate Justices. Buffalo: 1850.

This report did not reach us in time to be noticed in our August number, but we now take pleasure in bearing testimony to the elevated standing of Professor White, of the Buffalo Medical School, under the developments of this trial. We have already expressed our sentiments, in tolerably plain terms, on the subject of the persecutions against Dr. White, which persecutions, we are well persuaded, had their origin in something else than a desire to advance the interests of medical teaching. There are, we presume, in the shadow of all medical schools, envious spirits who are ever ready to spring upon the reputation of teachers who have been more fortunate than themselves in gaining position and character, and these ignoble factionists will "level down," if they cannot "level up."

The District Attorney for the State, in his opening address, gave Dr. Horatio N. Loomis, an amount of notoriety that has probably satiated his thirst for fame, for sometime, and Justice Mullet gave him a portrait, in charging the jury, that is almost as accurate as a Daguerreotype. Loomis was acquitted, notwithstanding the strong charge against him by the Presiding Justice, but he will wear his laurels without causing any honorable mind to violate the tenth commandment. B.

